# Pulmonary Artery-Tracheal Fistula After Coil Implantation for Behcet’s Disease

**DOI:** 10.7759/cureus.11036

**Published:** 2020-10-19

**Authors:** Dwayne M Hansen, Cornelius Dyke

**Affiliations:** 1 Surgery, University of North Dakota, Grand Forks, USA; 2 Cardiothoracic Surgery, Sanford Medical Center, Fargo, USA

**Keywords:** behcet’s syndrome, pulmonary artery - tracheal fistula, pulmonary artery aneurysm, coil embolization, coil migration, tracheal foreign body

## Abstract

Behcet’s disease (BD) is a rare autoimmune disorder that results in diffuse full-thickness vasculitis. Pulmonary artery aneurysms (PAAs) and hemoptysis are known complications of this disease process, with high morbidity and mortality for affected patients. Although medical, endovascular, and surgical treatment strategies have all been described in the literature, there are little data to describe the long-term outcomes of these various treatment modalities and there continues to be a lack of clearly defined algorithms for the management of these patients. We report a case of PAA in the setting of BD who was treated over the course of many years with medical therapy and coil embolization but ultimately failed treatment, sustained a complication of coil erosion and migration into the trachea twice, and required surgical lobectomy for definitive management. We discuss an algorithm for the management of patients with BD who have PAAs.

## Introduction

Behcet’s disease (BD) is an autoimmune disorder of unknown etiology characterized by diffuse vasculitis with full-thickness inflammation of affected vessels [1]. Pulmonary artery aneurysms (PAAs) are a known complication of BD and treatment options include medical, surgical, and endovascular techniques, including coil and glue embolization. The complication of coil migration after embolization has been reported; however, tracheal migration and pulmonary artery-tracheal fistula are rare. Additionally, optimal management of PAAs and their complications is unclear. In this report, we present a case of recurrent coil migration from a PAA into the trachea of a patient with BD and discuss our perspective on the management of this rare entity.

## Case presentation

**Figure 1:**
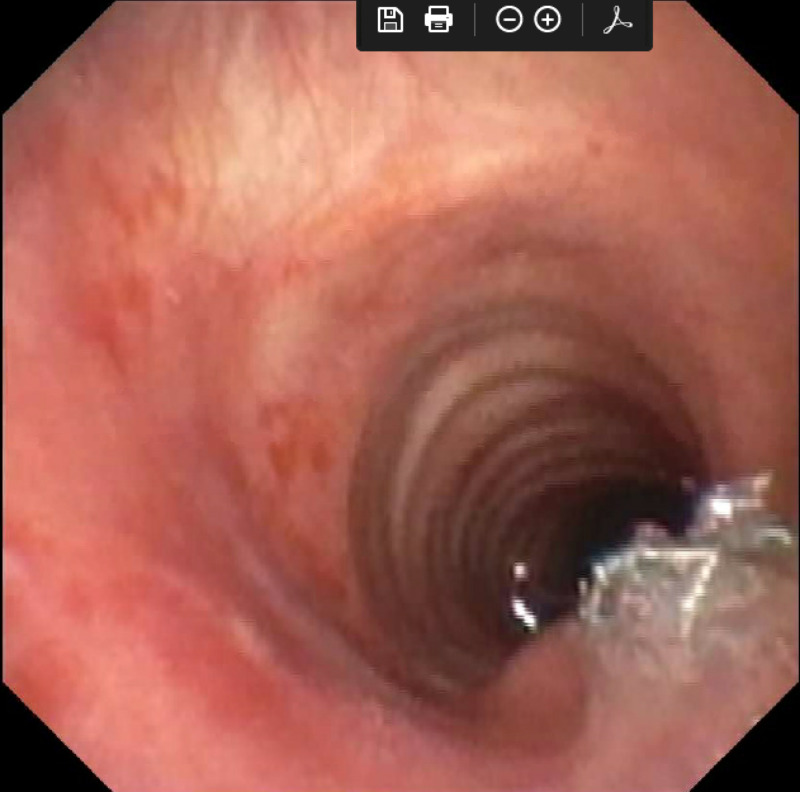
Metallic foreign body within the subglottic region.

**Figure 2:**
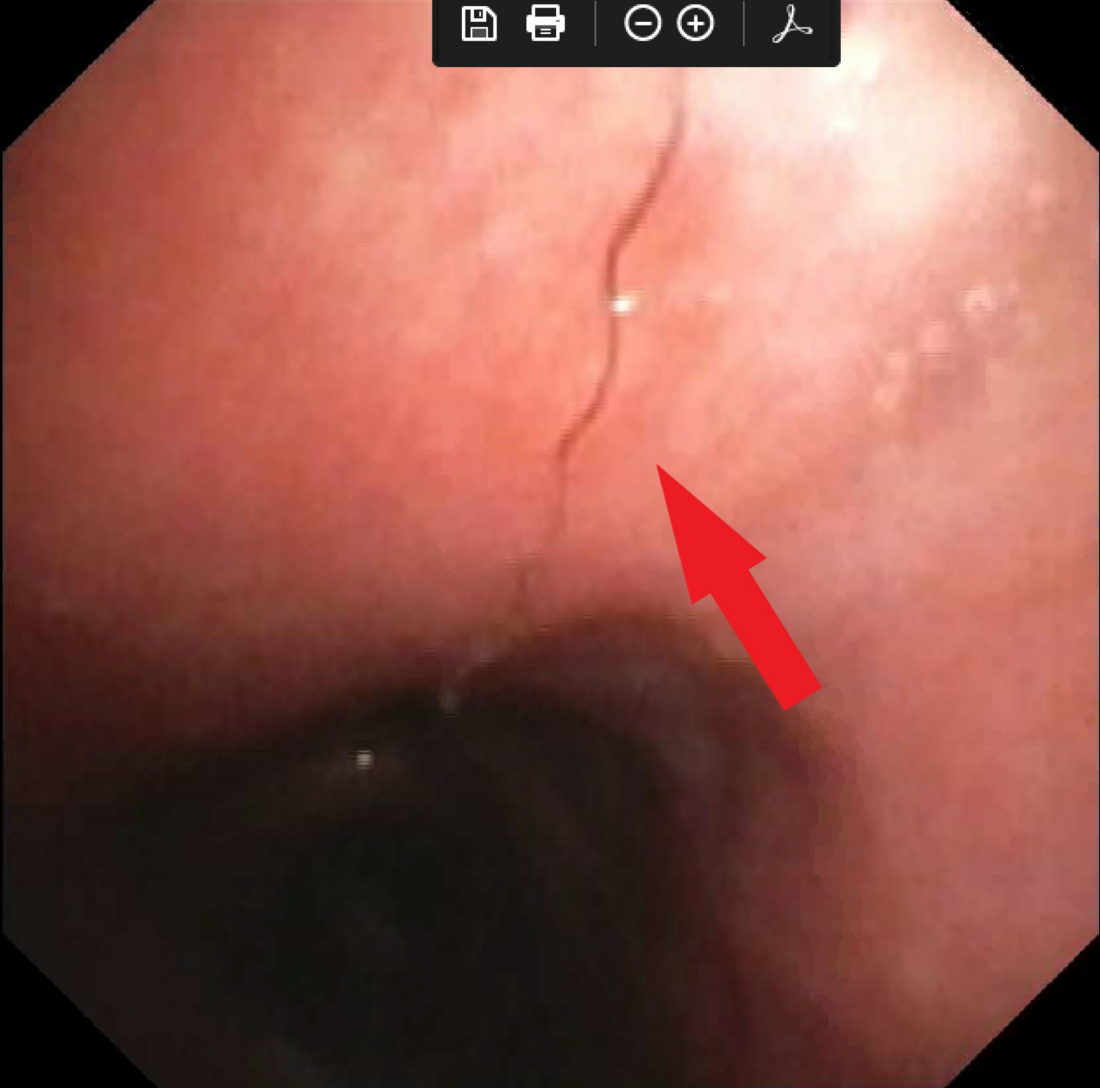
Metallic foreign body within the right main bronchus, which was followed to the right lower lobe.

**Figure 3:**
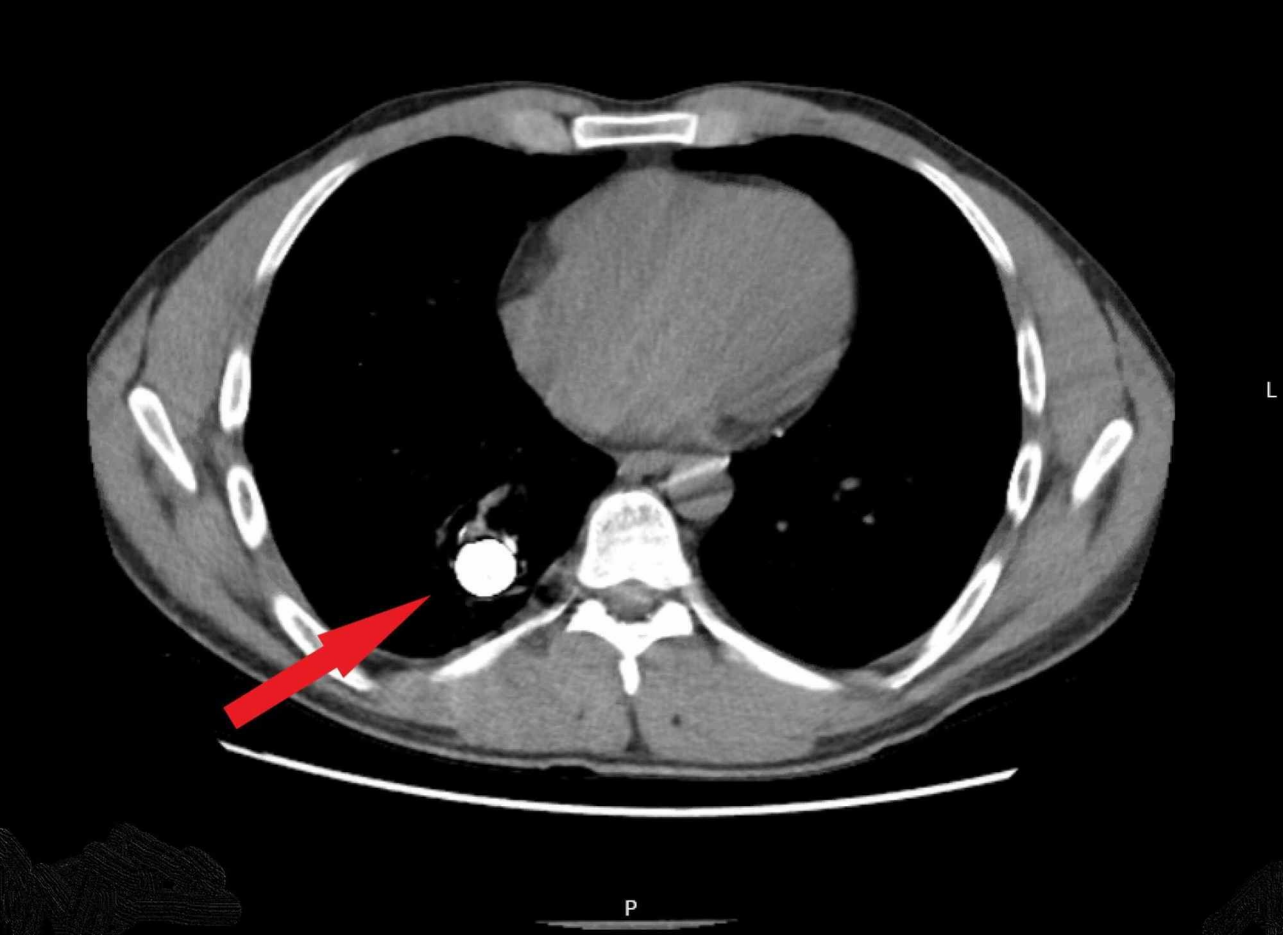
CT of the chest showing residual coil within the right lower lobe pulmonary artery aneurysm.

Our patient was a 29-year-old male of Middle Eastern origin with a known history of BD. He had been treated for many years with various immunosuppressive agents. Six years prior to our encounter, he developed hemoptysis and was diagnosed with a 2.5-cm PAA in the right lower lobe. He underwent coil embolization of the PAA and initially did well. Two years later, he developed recurrent hemoptysis with expectoration of a thin metallic wire. Bronchoscopy revealed a wire foreign body in the trachea extending into the right lower lobe bronchus. Traction on the wire allowed removal of additional several centimeters of wire until none was visible, and his hemoptysis resolved. He was well for an additional four years until he presented to us with recurrent severe cough and hemoptysis. Bronchoscopy again revealed metallic wire within the trachea and right lower lobe bronchus (Figures 1, 2). Chest CT revealed a large amount coil still within the PAA (Figure 3). After discussing these findings with the patient, he elected to undergo a right lower lobectomy with resection of the PAA and coil. Intra-operative bronchoscopy revealed a small fragment of residual wire within the right lower lobe bronchus, which was removed. His recovery was uneventful.

## Discussion

BD is an autoimmune disorder of unknown etiology. The trademark of BD is a systemic vasculitis with neutrophil infiltration and full-thickness inflammation of arteries and veins of all sizes [1,2]. Inflammation leads to perivascular edema and endothelial dysfunction, which alters vascular elasticity and promotes intravascular thrombosis and aneurysm formation [3]. The classic presentation of BD is that of recurrent uveitis, aphthous ulcers, and genital ulcerations; however, there is a constellation of symptoms that can occur and affect nearly every organ system.

It is estimated that 1-18% of patients with BD develop PAAs with hemoptysis, which can be massive [4]. Hemoptysis is an ominous presentation of BD, with early studies suggesting a one-year mortality of up to 50% [5]. Despite this significant risk, no consensus exists for the management of PAAs in patients with BD. Immunosuppressive therapy alone can lead to regression of symptoms and improvement of PAAs in up to 76% of patients and is often administered concurrently with surgical or endovascular techniques [6]. Cyclophosphamide and prednisone are frequently used first; if symptoms persist, other medications such as infliximab, azathioprine, colchicine, or methotrexate may be used [6-8]. Endovascular techniques or surgery are usually considered with failure of medical management.

Endovascular embolization has been described as an effective treatment for the complication of hemoptysis in patients with PAAs. Cyanoacrylate glue has been used to treat hemoptysis in patients with tuberculosis, with good medium-term results [9]. Other endovascular techniques such as coil implantation to promote thrombosis within the aneurysm have also been used with varying success. Although embolization is 80-90% effective at controlling acute hemoptysis from all causes, the three-year recurrence of hemoptysis is nearly 45% [10]. Complications of coil implantation such as erosion and migration have been reported years after intervention, and migration into the airway is uncommon but has been described [11-13]. A literature review in 2013 evaluated 107 reports of PAA treatment in patients with BD and evaluated medical, endovascular, and surgical management in a total of 173 patients. Conservatively treated patients were prone to progression, embolization was associated with a higher risk of recurrence and reintervention, and surgery was associated with the highest overall and early mortality rates especially when performed urgently [6]. The true incidence of coil migration, erosion, or long-term recurrence in patients with PAAs due to BD remains unknown.

Surgery has been used to treat PAAs [14]. Proximal aneurysms may be reconstructed with aneurysmorrhaphy [15,16]. Lobectomy seems to be the most commonly described approach for patients with PAAs and BD [14]. Surgical management carries a higher morbidity and mortality in the acute setting for patients with massive hemoptysis. However, when performed on an elective basis, after aggressive medical management, the outcomes for patients with BD and PAAs treated with resection are good [14,17]. There are no recommendations for the management of endovascular complications such as coil migration to the trachea. Recurrent coil migration, as in our patient, is even more unusual. While endovascular techniques are clearly useful for the initial management of hemoptysis, our experience suggests that surgical resection should be considered at the time of endovascular treatment failure. Surgery may also have benefit over endovascular treatment for patients who do not require urgent intervention and can be managed outpatient with aggressive immunosuppression and then elective surgical resection. Elective surgical resection can be considered even after successful endovascular treatment given the risk of recurrent symptoms.

**Figure 4:**
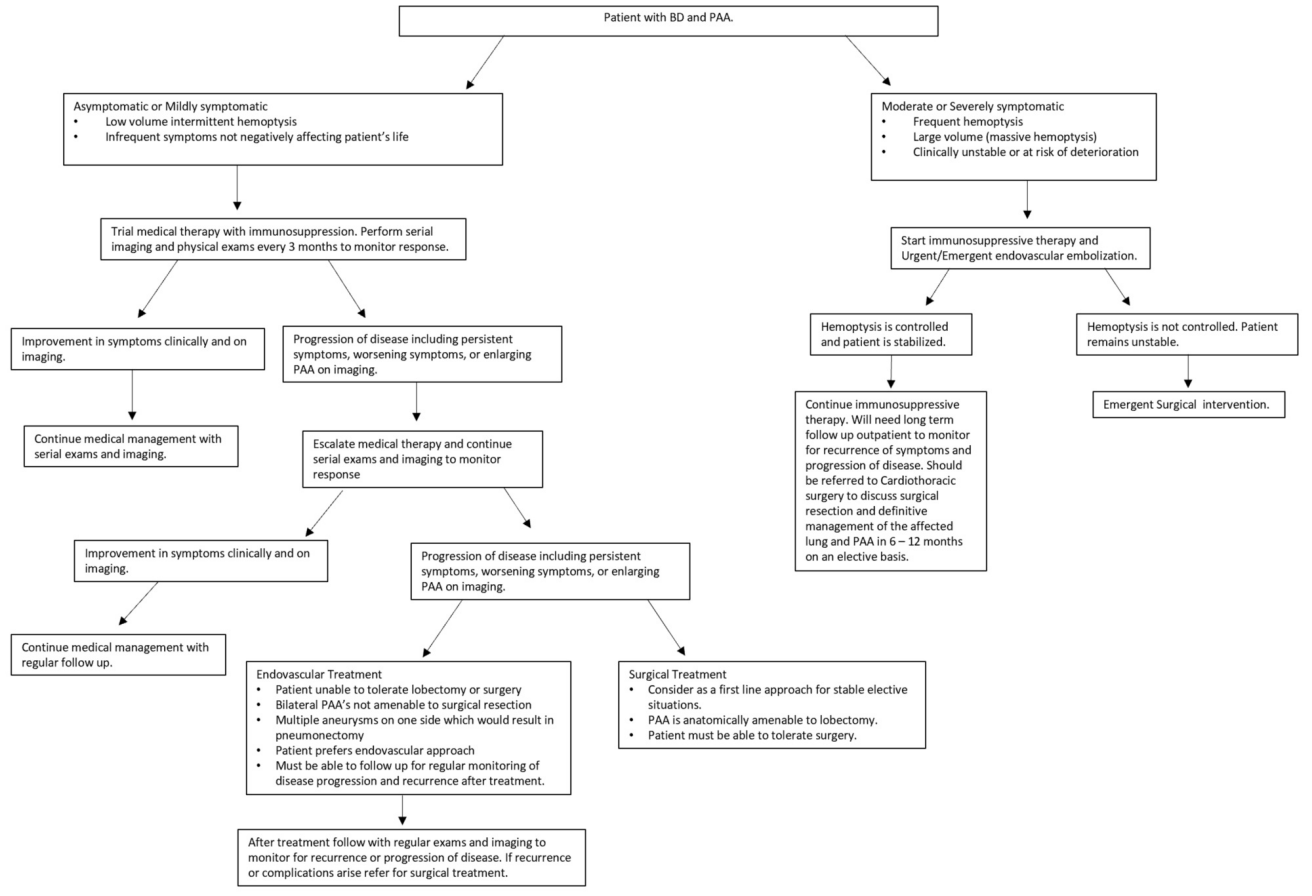
Treatment algorithm for patients with PAAs in the setting of BD. PPA, pulmonary artery aneurysms; BD, Behcet's disease

Based on our case and literature review, we have organized a treatment algorithm to help guide our management of this rare entity should we encounter it again (Figure 4). Additional studies are needed to validate our algorithm; however, it serves as a starting point for further discussion and research.

## Conclusions

BD is a rare entity that predisposes patients to developing symptomatic PAAs. Although the literature describes medical, endovascular, and surgical treatments, there are no defined treatment algorithms. Patients with BD and PAAs treated with embolization may be at increased risk of late complications such as coil erosion and migration given the underlying inflammatory process. Medical therapy is a cornerstone of treatment, and the literature suggests that all patients, if able, should be treated with immunosuppression. We feel that endovascular and surgical treatment should be considered early. If the PAA is anatomically amenable to lobectomy, elective surgical resection has low risk and is a durable solution. In the setting of massive hemoptysis, endovascular embolization is an emergent solution, and surgical resection can be reserved for treatment failure, recognizing that urgent surgery carries increased morbidity and mortality. For any recurrent disease, hemoptysis, or complications associated with endovascular embolization, surgical resection may be a more effective treatment. BD is a complex disorder that requires a multi-team approach with attentive medical and surgical management.
